# Tuning Redox State and Ionic Transfers of Mg/Fe-Layered Double Hydroxide Nanosheets by Electrochemical and Electrogravimetric Methods

**DOI:** 10.3390/nano10091832

**Published:** 2020-09-14

**Authors:** Elise Duquesne, Stéphanie Betelu, Alain Seron, Ioannis Ignatiadis, Hubert Perrot, Catherine Debiemme-Chouvy

**Affiliations:** 1 Laboratoire Interfaces et Systèmes Électrochimiques (UMR8235), Sorbonne Université, CNRS, 4 Place Jussieu, 75005 Paris, France; elise.duquesne59@gmail.com (E.D.); hubert.perrot@sorbonne-universite.fr (H.P.); 2 BRGM, French Geological Survey, 3 Avenue Claude Guillemin, 45000 Orléans, France; a.seron@brgm.fr (A.S.); i.ignatiadis@brgm.fr (I.I.)

**Keywords:** layered double hydroxide (LDH), electrochemical quartz crystal microbalance (EQCM), redox reactions, ionic exchanges

## Abstract

Studying the electrogravimetric behavior of Mg/Fe-layered double hydroxide (LDH) nanoparticles with an electrochemical quartz crystal microbalance demonstrates its pseudocapacitance properties of mix cation and anion exchanger. The electrochemical control of the oxidation state of iron constituting the layered sheets allowed anion intercalation/deintercalation into the LDH interlayer space. Concomitantly, in agreement with the pH of zero point of net charge of the Mg/Fe-LDH, the interfacial pH increase via catalyzed hydrogen evolution reaction allows cation electroadsorption onto the external surfaces of the nanoplatelets.

## 1. Introduction

Layered double hydroxide (LDH) materials consist in a stacking of positively charged brucite-like layers, due to the substitution of some divalent cations, M(II), by trivalent cations, M(III). The charge compensation is managed by hydrated anions A^n−^ intercalated into the interlayer space. This leads to the general formula: [M(II)_1−x_M(III)_x_(OH)_2_]^x+^[A^n−^]_x/n_, mH_2_O, providing a high capacity for anion exchange [1,2,3,4].

It is well established [5] that a reversible anion transfer between the interlayer spaces and the electrolyte is induced by tuning the redox state of LDHs according to:

(1) $$y\;\left[\mathrm{LDH}\;:\;M(\mathrm{II})\right]\;+\;\;\frac{y}{n}A^{n-\;}{}^{}↔\;y\;\left[\mathrm{LDH}\;:\;M(\mathrm{III})\;:\;\frac{1}{n}\;A^{n-}\right]{+\;y\;e}^{-}$$

where electrons are displaced within the layer via a hopping mechanism. The ion uptake/release occurring during redox processes can be monitored with suitable in situ coupled methods, i.e., combining structural, electrochemical and electrogravimetric techniques.

For progress in broad applications where layered materials are employed, for instance in water treatment [6,7,8,9,10], water splitting [11,12,13] or energy storage [14,15], the reversibility of the ion transfer between the electrolyte and the material merits further investigation. Only a few studies [5,16,17] have explored the ion transfer phenomenon and its reversibility related to the repetitive oxidation/reduction of electroactive cations within the layers for the most conducting LDHs (Co/Ni-LDH, Ni/Al-LDH, Mg/Al/Fe-LDH, Fe/Fe-LDH) by probing the mass variation with electrochemical quartz crystal microbalance (EQCM) [5,17,18,19,20,21]. To the best of our knowledge, no study has been conducted yet on Mg/Fe-LDH.

This communication reports on a study conducted for the first time by EQCM to investigate the ion transfer phenomena of Mg/Fe-LDH. It has been demonstrated that Fe sites are reversibly switched, under polarization, between (+III) and (+II) oxidation state inducing anion intercalation or deintercalation. The pH of zero point of net charge [22], pH_ZPNC_ (pH resulting from the cancellation of the positive charge originating from the chemical composition of the LDH and the variable charge resulting from the protonation/deprotonation of the hydroxyls of the outlayers) and the pH increase at the material/electrolyte interface due to the release of hydroxyl ions during water electroreduction play a key role on the cation electroadsorption onto the nanosheets.

## 2. Materials and Methods

LDH particle synthesis consists in co-precipitation of Mg/Fe by increasing pH of an aqueous solution containing 0.0625 mol/L Mg(NO_3_)_2_ + 0.03125 mol/L Fe(NO_3_)_3_, thermal maturation, purification via centrifugation and finally nitrate exchange by carbonates via a dialysis, leading to Mg/Fe-CO_3_^2−^ particles [23,24]. The resulting suspension was drop casted (10 µL) onto the gold electrode of a quartz resonator (operating at 9 MHz, AWS Sensors, Spain) and dried at room temperature.

After drying the slurry at 70 °C for 48 h, the LDH was characterized by powder X-ray diffraction with a Empyrean diffractometer (Malvern Panalytical, Almelo, The Netherlands), using a Cu Kα radiation (λ = 1.541 Å) operating at 45 kV and 40 mA at room temperature. The diffractometer is equipped with a detector operating in scanning line mode using 255 channels. The scans were recorded from 4° to 84° (2θ) with a step of 0.026° and an acquisition time of 1200 s per step.

The morphology of the film dried onto gold electrode was examined under a field emission gun scanning electron microscope (FEG-SEM) Ultra55 Zeiss (Oberkochen, Germany) operating at 10 keV.

The LDH Mg/Fe ratio was deduced from the atomic absorption spectroscopy analysis (Spectra AA 220 FS VARIAN 122 instrument) performed on samples digested in nitric acid solution (1 mol/L).

EQCM measurements were conducted in a 3-electrode cell configuration using an Autolab PGSTAT302 potentiostat (Metrohm, Switzerland) coupled with a laboratory-made QCM device. A gold patterned quartz substrate coated with the Mg/Fe-LDH was employed as working electrode (S = 0.2 cm^2^), a Pt grid as a counter electrode and a silver chloride electrode with KCl 3 mol/L (Ag/Ag^+^) as a reference electrode. The aqueous electrolyte was deaerated with nitrogen gas. Frequency change, ∆f, of the quartz crystal resonator was monitored during cyclic voltammetry measurements. Then, ∆f was converted into mass change, ∆m, by using the Sauerbrey equation [25]:

∆f = −k_s_ ∆m (2)

where k_s_ is the sensitivity factor (the experimental value, 16.31 × 10^7^ Hz·g^−1^·cm^2^ for 9 MHz, was obtained by copper electrodeposition on gold patterned quartz substrate [26]).

## 3. Results and Discussion

### 3.1. Structural and Morphological Characterization of the LDH

**Figure 1:**
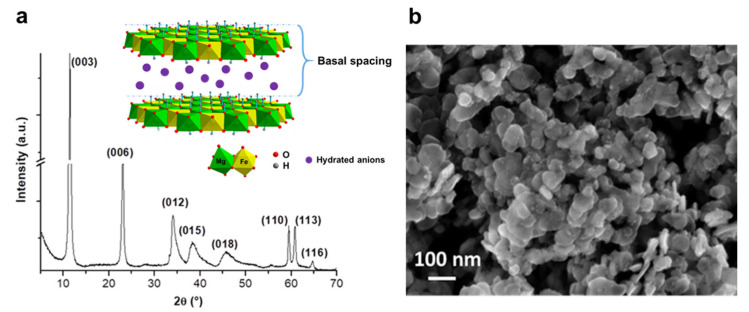
(**a**) X-ray powder diffraction pattern of the nano-Mg/Fe-layered double hydroxide (LDH) and structure of the Mg/Fe LDH, (**b**) SEM micrograph of the Mg/Fe-LDH coated gold electrode. S = 0.2 cm^2^.

The powder X-ray diffraction pattern (Figure 1a) shows a characteristic LDH structure (see insert Figure 1a) [27]. The basal spacing (d = 7.74 Å) was calculated by Bragg law equation from (003) reflection.

SEM micrograph (Figure 1b) reveals that the LDH is made of nanoplatelets whose size is in the range from 50 up to 100 nm with an average size about 80 nm, implying a high surface-to-volume ratio. The molar ratio Mg/Fe = 2 was confirmed with atomic absorption spectroscopy.

### 3.2. Polarization Induced Ionic Transfers

EQCM was conducted on the gold electrode of quartz resonators uncoated and coated with a thin film of Mg/Fe-LDH. The measurements were recorded at 25 mV·s^−1^ in a 0.1 mol/L carbonate-buffered solution whose pH = 10.8, similar to the pH_ZPNC_ of the LDH, which was calculated to be 10.6 [22]. Therefore, one could consider that initially the LDH particles are globally uncharged.

The behavior of the Mg/Fe-CO_3_^2−^ deposit was successively investigated in three different potential ranges, i.e., [−1.8; −0.3] V, [−1.2; −0.3] V and [−1.5; −0.3] V. Actually, only the cathodic limit was changed in order to highlight the reduction of H_2_O molecules present in the intercalated spacing of the LDH and also to vary the pH at the LDH/solution interface to evidence the sorption of cation onto the surface of the LDH nanoplatelets. Finally, the electrode was rinsed with distilled water, dried at room temperature, and investigated again in the initial potential range [−1.8; −0.3] V. The results were compared to the behavior of a bare gold-patterned resonator.

#### 3.2.1. EQCM in the [−1.8; −0.3] V Range

**Figure 2:**
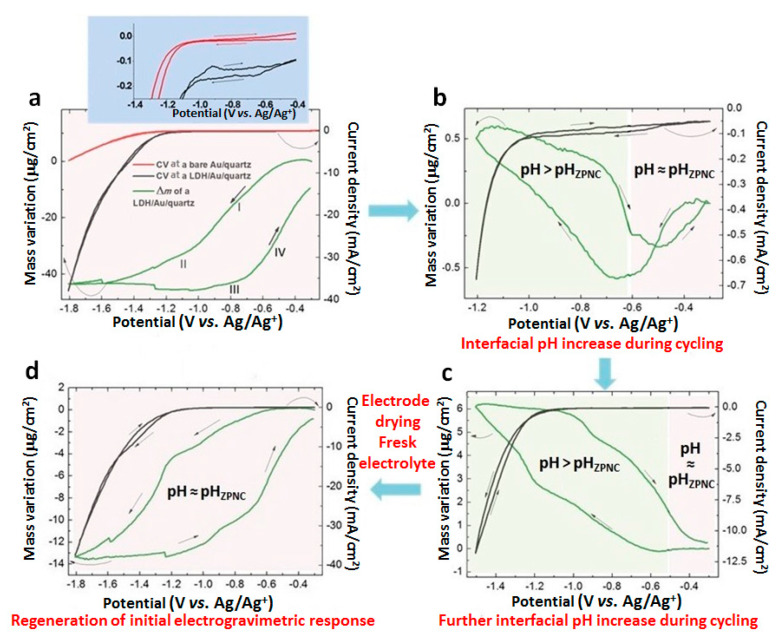
Representative electrochemical quartz crystal microbalance (EQCM) measurements of a gold electrode coated with Mg/Fe-LDH successively cycled in different potential ranges in deaerated 0.1 mol/L carbonate solution (pH = 10.8): (**a**) [−1.8; −0.3] V, (**b**) [−1.2; −0.3] V, (**c**) [−1.5; −0.3] V, (**d**) [−1.8; −0.3] V after drying the electrode in air for 12 h.

Figure 2a shows the current-potential curve (in red) at a bare gold-patterned resonator in the range [−1.8; −0.3], V where water reduction starts below −1.2 V.

In comparison, below −1.2 V for the LDH-coated electrode (black curve), the higher cathodic current reveals the catalysis of the reduction of H_2_O from the bulk of the solution. In addition, between −0.3 V and −1 V (Figure 2a, inset), the negative current during the cathodic and anodic sweep and especially the pseudo-plateau below −0.6 V indicate a diffusion-limited reaction that can be ascribed to reduction of interlayered water molecules. In Figure 2a, the mass loss during the cathodic sweep (slope I, from −0.6 V to −1 V) confirms this assumption. This mass variation is attributed to the expulsion of the electrogenerated OH^-^ from the interlayer space due to negative charges in excess. Inversely, during the anodic sweep (slope IV, from −0.7 V to −0.3 V) the mass gain could be due to the lower kinetics of the water reduction compared to that of the water molecule re-intercalation by diffusion.

The electrochemical reoxidation of Fe(II) is observed around −0.95 V, Fe(III) reduction being masked by the bulk water reduction. Due to Fe(III) reduction, the charge of the layers decreases which is responsible for anion deintercalation from the interlayer space. This phenomenon is confirmed by the mass loss from −1.0 V to −1.6 V (slope II in Figure 2a). Inversely, the mass gain between −1.0 V and −0.7 V (slope III in Figure 2a) is induced by the Fe(II) oxidation and is most likely due to the intercalation of CO_3_^2−^_,_ which has the strongest affinity for LDH [28] following:

[Mg_4_^II^Fe_2_^III^(OH)_16_]^2+^[CO_3_]^2−^, mH_2_O + (x + y) e^−^ ⇆
[Mg_4_^II^Fe_2−x_^III^Fe_x_^II^(OH)_16_]^(2−x)+^[(CO_3_^2−^)_1–0.5x_]^(2−x)−^,(m−y)H_2_O + 0.5x CO_3_^2−^ + 0.5y H_2_ + y OH^−^ (3)

This result is in agreement with the results of Duquesne et al. [17], who investigated electrochemical activity of Ni^II^/Ni^III^ redox couple within Ni/Fe LDHs in NaOH 1 mol/L solution. They showed that the nickel oxidation within the LDH leads to OH^-^ intercalation and H_2_O de-intercalation.

#### 3.2.2. EQCM in the [−1.2; −0.3] V Range

In order to avoid bulk water reduction and investigate the reduction of the water molecules present in the interlayer spaces, the cathodic limit was reduced to −1.2 V after the 7th cycle, for 16 cycles (Figure 2b). As described previously between −0.3 V and −0.6 V, the cathodic mass loss and anodic mass gain are in agreement with the dehydration/rehydration of the LDH interlayer space. Between −0.6 V and −1.2 V, the mass variations, increasing at each incremented cycle, are attributed to a phenomenon that becomes preponderant. The continuous hydrogen evolution reaction, while cycling, increases the OH^−^ amount at the vicinity of the electrode. This pH increase is responsible for the deprotonation of some Metal-OH functions of the LDH external layers since the pH_ZPNC_ is exceeded. The reduction of H_2_O in the intercalated spaces is sufficient to drive sodium ion electroadsorption/desorption during the cathodic/anodic sweep. In the potential domain where the interfacial pH is superior to pH_ZPNC_ (in green in Figure 2b), the mass variation, 1 µg/cm^2^, corresponds to 2.6 × 10^16^ ions/cm^2^, taking into account the geometric surface of the electrode.

#### 3.2.3. EQCM in the [−1.5; −0.3] V Range

Continuing the electrochemical cycling with a higher cathodic limit, −1.5 V, during 9 cycles (Figure 2c) allows the reduction of more bulk water molecules. Indeed, the cathodic current limit is higher, 12 mA/cm^2^ (Figure 2c) vs. 0.65 mA/cm^2^ (Figure 2b). The interfacial pH increase leads to an enhanced deprotonation of the Metal-OH functions of the external layers of the LDH nanoparticles and reversible cation electroadsorption onto the surface of the LDH nanosheets in larger quantity than previously observed in potential range [−1.2; −0.3] V (Figure 2b) (6 µg/cm^2^ vs. 1 µg/cm^2^). Under these conditions, cation electroadsorption/desorption dominates the LDH electrogravimetric response following:

[Mg_4_^II^Fe_2-x_^III^Fe_x_^II^(OH)_16_]^(2-x)+^[(CO_3_^2−^)_1–0.5x_]^(2−x)−^,(m−y)H_2_O + y OH^−^ + z Na^+^ ⇆
[Mg_4_^II^Fe_2−x_^III^Fe_x_^II^(OH)_16−z_ (ONa)_z_]^(2−x)+^[(CO_3_^2−^)_1−0.5x_]^(2−x)−^,(m−y) H_2_O + (y−z) OH^−^ + z H_2_O (4)

Therefore Na^+^ sorption and desorption result from the interfacial pH variation induced by water reduction reaction. Thus, during the cathodic scan, water reduction causes an increase of the interfacial pH and allows cation sorption to the negatively charged external surface of the LDH nanoplatelets. Whereas during the backward anodic scan, the current progressively decreases and the interfacial pH becomes equal to that of the solution bulk, leading to cation desorption from the external surface of the LDH nanoplatelets.

#### 3.2.4. EQCM in the [−1.8; −0.3] V Range–Regeneration of the Initial Mg/Fe-LDH

The electrode was then rinsed with bi-distilled water and dried at room temperature. It was reused in a fresh electrolyte under the same initial conditions, i.e., in the potential range [−1.8; −0.3] V (Figure 2d). A similar electrogravimetric response is observed (Figure 2a) because of the restoration of the initial pH at the interface. The decrease in mass variation is probably owing to the loss of material during the rinsing.

## 4. Conclusions

The ionic exchanges, occurring in response to the monitoring of the redox state of a thin film formed by Mg/Fe-LDH nanoplatelets, were investigated by EQCM and clearly illustrate pseudo-capacitance properties of mix (cation and anion) exchanger. The concomitant electrochemical control of (i) the chemical and physical conditions at the interface, (ii) the oxidation state of the cations constituting the layered sheets of the LDH, and (iii) the zero point of net charge allowed anion intercalation/deintercalation into the interlayer space and cation sorption/desorption onto the external sheets at the interface. For a better insight, further electrogravimetric and structural investigations are in progress notably by coupling EQCM and in situ X-ray diffraction.
